# Early weight loss in parkinsonism predicts poor outcomes: Evidence from an incident cohort study

**DOI:** 10.1212/WNL.0000000000004691

**Published:** 2017-11-28

**Authors:** Kirsten Cumming, Angus D. Macleod, Phyo K. Myint, Carl E. Counsell

**Affiliations:** From the School of Medicine (K.C.), Medical Sciences & Nutrition, and Chronic Disease Research Group (A.D.M., C.E.C.) and Ageing Clinical & Experimental Research Team (P.K.M.), Institute of Applied Health Sciences, University of Aberdeen, Scotland.

## Abstract

**Objective:**

To compare weight change over time in patients with Parkinson disease (PD), those with atypical parkinsonism, and matched controls; to identify baseline factors that influence weight loss in parkinsonism; and to examine whether it predicts poor outcome.

**Methods:**

We analyzed data from the Parkinsonism Incidence in North-East Scotland (PINE) study, an incident, population-based prospective cohort of parkinsonian patients and age- and sex-matched controls with annual follow-up. Mixed-model analysis described weight change in patients with PD, those with atypical parkinsonism, and controls. Baseline determinants of sustained clinically significant weight loss (>5% loss from baseline) and associations between early sustained weight loss and death, dementia, and dependency in parkinsonism were studied with Cox regression.

**Results:**

A total of 515 participants (240 controls, 187 with PD, 88 with atypical parkinsonism) were followed up for a median of 5 years. At diagnosis, atypical parkinsonian patients had lower body weights than patients with PD, who were lighter than controls. Patients with PD lost weight more rapidly than controls, and weight loss was most rapid in atypical parkinsonism. After multivariable adjustment for potential confounders, only age was independently associated with sustained clinically significant weight loss (hazard ratio [HR] for 10-year age increase 1.83, 95% confidence interval [CI] 1.44–2.32). Weight loss occurring within 1 year of diagnosis was independently associated with increased risk of dependency (HR 2.11, 95% CI 1.00–4.42), dementia (HR 3.23, 95% CI 1.40–7.44), and death (HR 2.23, 95% CI 1.46–3.41).

**Conclusion:**

Weight loss occurs in early parkinsonism and is greater in atypical parkinsonism than in PD. Early weight loss in parkinsonism has prognostic significance, and targeted dietary interventions to prevent it may improve long-term outcomes.

Previous studies have shown that weight loss is common in Parkinson disease (PD),^1,2^ is not explained solely by reduced nutritional intake or increased energy expenditure,^3^ and may be associated with worse outcomes.^4–9^

However, these previous studies have generally been based on small, highly selected cohorts (frequently relatively young-onset patients) with limited or no follow-up.^1,3–5,8^ Only 2 studies have compared the degree of weight loss and its early risk factors in PD,^5,10^ but none has compared PD with other atypical parkinsonian syndromes, compared patients with PD with controls over long-term follow-up from diagnosis in an unselected incident cohort of patients, or assessed the effect of early weight loss on long-term outcomes. Hence, uncertainty remains in the degree, causes, and relevance of weight loss in PD and other parkinsonian syndromes.

We aimed to compare weight change over time in parkinsonian patients with age- and sex-matched controls, to determine which factors measured at diagnosis are associated with developing sustained clinically significant weight loss in parkinsonism, and to evaluate the effect of early significant weight loss in parkinsonism on clinical outcomes.

## METHODS

### Study design.

The Parkinsonism Incidence in North-East Scotland (PINE) study is a population-based incident cohort of parkinsonism with annual prospective lifelong follow-up.^11,12^ The study design and recruitment methods were previously described.^11,12^ In brief, multiple ascertainment strategies were used to identify all new diagnoses of degenerative or vascular parkinsonism occurring in a population of ≈300,000 during 2 incidence periods, 2002 to 2004 and 2006 to 2009. Patients were invited to consent to lifelong follow-up. Age- and sex-matched controls were recruited from the same general practice as each recruited patient. Patients and controls had annual assessment including clinical history and examination, review of medical case notes, and various assessment scales. Body weight was measured annually with regularly calibrated hospital scales.

Patients' diagnoses were reviewed annually by a neurologist with a special interest in movement disorders or by supervised specialist trainees in neurology or geriatric medicine. Clinical diagnoses were guided by appropriate research criteria, e.g., the Parkinson’s UK Brain Bank criteria.^11,13^ Controls and parkinsonian patients with at least 2 weight measurements throughout clinical follow-up were included in these analyses. The parkinsonian group was analyzed separately as those with PD and those with atypical parkinsonism.

### Standard protocol approvals, registrations, and patient consents.

Ethics approval was obtained from the Grampian Research Ethics Committee and the Multi-Centre Research Ethics Committee for Scotland. All participants gave written informed consent.

### Weight data.

The PINE study database was screened for missing annual weight data. When those data were missing, available sources (medical and nursing notes, GP summaries) were examined for weights measured within 6 months of the date of the visit.

In this study, clinically significant weight loss was defined as loss of >5% of baseline body weight as previously described.^14^ Follow-up weight measurements were screened to identify those participants with sustained clinically significant weight loss (i.e., weight loss of >5% of the baseline weight, with all subsequent weight measurements being >5% less than baseline weight).

### Outcome data.

We selected 3 clinically relevant outcomes of interest: dependency (need for help with basic activities of daily living), dementia, and death. Dependency was defined as a Schwab and England score <80.^15^ Dementia was diagnosed with a DSM-IV definition based on clinical interview supplemented by cognitive testing with the Mini-Mental State Examination and Mini-Mental Parkinson's.^16^ All participants were flagged with the NHS central register for regular notifications of deaths.

### Statistical analysis.

#### Description of weight change in patients with PD, those with atypical parkinsonism, and controls.

We used a repeated-measures linear mixed model to describe weight change over time. Annual weight measurements were set as the dependent variable. Age, diagnostic group (PD, atypical parkinsonism, controls), and years of follow-up were entered as main fixed-effect factors, and interactions between each fixed effect and weight measurements were also included. A first-order autoregressive covariance structure was assumed. Yearly estimates of mean weight change were calculated from marginal means from the model, and the significance of the interaction between diagnosis and years of follow-up was used to determine whether the change in weight over time was different between diagnostic groups. To determine whether weight change over time was different between PD and atypical parkinsonism, we repeated the model excluding controls and tested the significance of the interaction between PD/atypical parkinsonism diagnosis and year of follow-up. Similarly, the model was repeated for controls and patients with PD only.

#### Determinants of weight loss in parkinsonism.

Baseline determinants (factors measured at the first [diagnostic] visit) of sustained significant weight loss in parkinsonian patients were investigated with multivariable Cox regression. The following variables were considered for entry in the model: age; sex; PD vs atypical parkinsonism; baseline Charlson comorbidity score^17^; presence of hallucinations; self-reported problems with swallowing; low mood and memory; dependency (Schwab and England score <80); dementia; Barthel Index; Mini-Mental State Examination; Parkinson's Disease Questionnaire 8; Unified Parkinson's Disease Rating Scale parts I, II, and III subscores; and Hoehn-Yahr score. Cases without clinically significant weight loss were censored at the date of the last available weight measurement. Univariable analyses of the baseline covariates were examined. Variables with statistical significance (*p* < 0.05) in the univariable analysis were included in a backward stepwise selection process, with *p* > 0.1 being the criterion for removal from the model to reduce the likelihood of chance findings in the models. Variables with >10% of missing data and variables that showed important collinearity with other variables were excluded. To avoid overfitting in the final models, we ensured that the ratio of events to variable was <10.^18^

#### Prognosis of clinically significant weight loss in parkinsonism.

Three separate multivariable Cox regression models were constructed to assess the associations between sustained clinically significant weight loss within the first year of diagnosis and dependency, dementia, and death. Patients who developed an outcome of interest within the first year of diagnosis were excluded from the analyses predicting that outcome. Losses to follow-up were censored at the date of last follow-up, and data were extracted for analysis on December 31, 2014.

The same variables and selection strategy for models assessing outcomes as described above were used for the model of time to sustained significant weight loss.

For each Cox model, the proportional hazards assumption was checked by visual inspection of the Kaplan-Meier survival plots. Complete-case analysis was performed for each Cox model; i.e., patients with missing data were removed from each model.

#### Sensitivity analysis.

We also performed a post hoc sensitivity analysis to examine whether excluding patients with vascular parkinsonism (which is not strictly neurodegenerative) from the atypical parkinsonism group altered our results.

We used Statistical Package for Social Sciences version 21.0 (SPSS Inc, Chicago, IL) and Stata version 12.1 (StataCorp, College Station, TX) to perform the analyses.

## RESULTS

**Figure 1:**
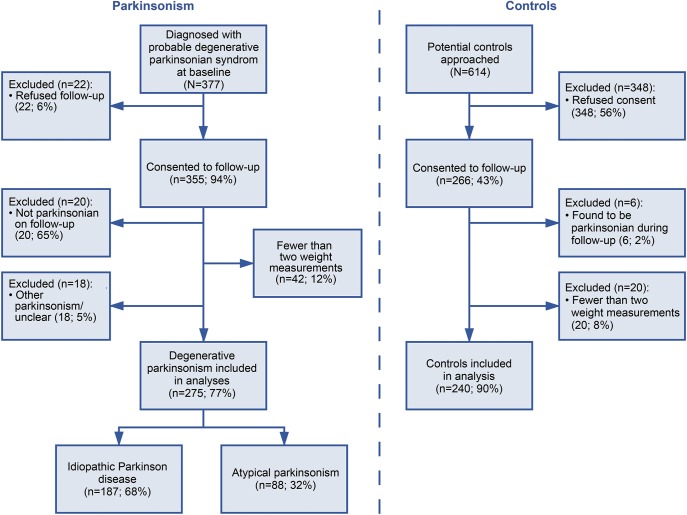
Study flowchart Flowchart of Parkinsonism Incidence in North-East Scotland (PINE) study participants.

Of 355 patients with parkinsonism and 266 controls in the PINE study, 187 with PD, 88 with atypical parkinsonism, and 240 controls were included. Reasons for exclusions are given in figure 1. The atypical parkinsonian group included those with corticobasal degeneration (n = 2), dementia with associated parkinsonism (n = 4), dementia with Lewy bodies (n = 26), multiple system atrophy (n = 14), progressive supranuclear palsy (n = 18), and vascular parkinsonism (n = 24). Patients and controls were followed up for up to 12 years (mean duration until death, loss to follow-up, or last follow-up 6.9 years in controls, 6.3 years in patients with PD, and 4.2 years in those with atypical parkinsonism). Only 1 patient and 1 control were lost to follow-up for reasons other than death.

### Description of weight change in patients with PD, those with atypical parkinsonism, and controls.

**Figure 2:**
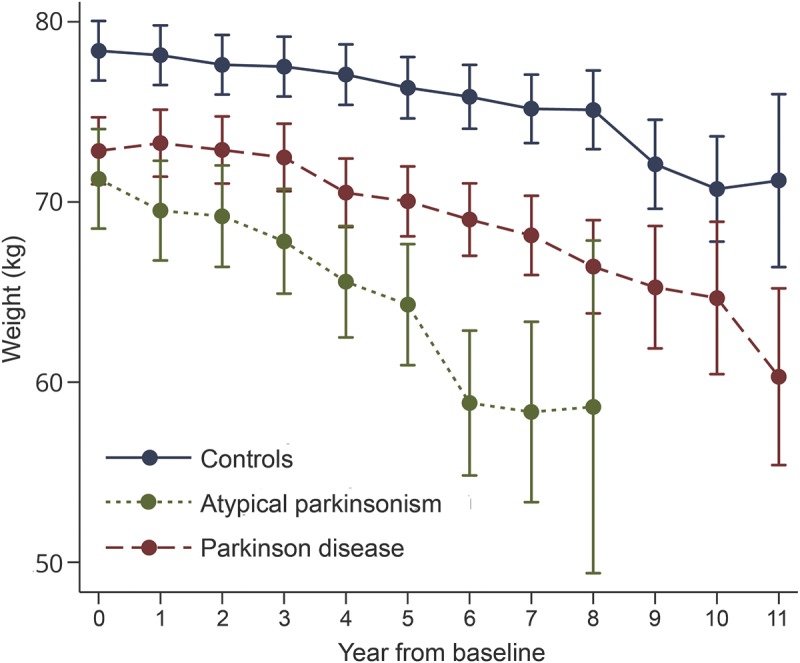
Weight change over time in controls and those with PD and atypical parkinsonian syndromes Adjusted yearly estimates of mean weight in kilograms from baseline (from patients' diagnosis and from control recruitment) in controls, patients with idiopathic PD, and those with atypical parkinsonian syndromes. Bars represent the 95% confidence intervals for each data point. PD = Parkinson disease.

Baseline characteristics and comparisons between individuals with and without sustained clinically significant weight loss are presented in table 1. On average, atypical parkinsonian patients were older than those with PD, and there were more men. At diagnosis, patients with PD and atypical parkinsonian patients were significantly lighter than controls by 5.6 and 7.1 kg, respectively, after adjustment for age and sex (*p* < 0.001). After diagnosis, weight reduced in all study groups (figure 2). Mean (SD) for the decrease in weight from baseline until the last follow-up visit was 3.2 (6.9), 4.1 (8.6), and 5.0 (7.6) kg in controls, patients with PD, and those with atypical parkinsonism, respectively. These data were not obviously skewed (median decreases 3, 4, and 5 kg). Patients (all with parkinsonism) lost weight more quickly than controls (*p* = 0.001 for the interaction between patient/control status and year). Patients with PD lost weight more quickly than controls (*p* = 0.02 for the interaction between controls/those with PD and year). Patients with atypical parkinsonism lost weight more quickly than those with PD (*p* = 0.001 for the interaction between PD/atypical parkinsonism and year). Sustained clinically significant weight loss within the first year of diagnosis was more common in patients with atypical parkinsonism (22%) than in those with PD (8%) and controls (8%) (*p* = 0.002, table 1).

**Table 1:**
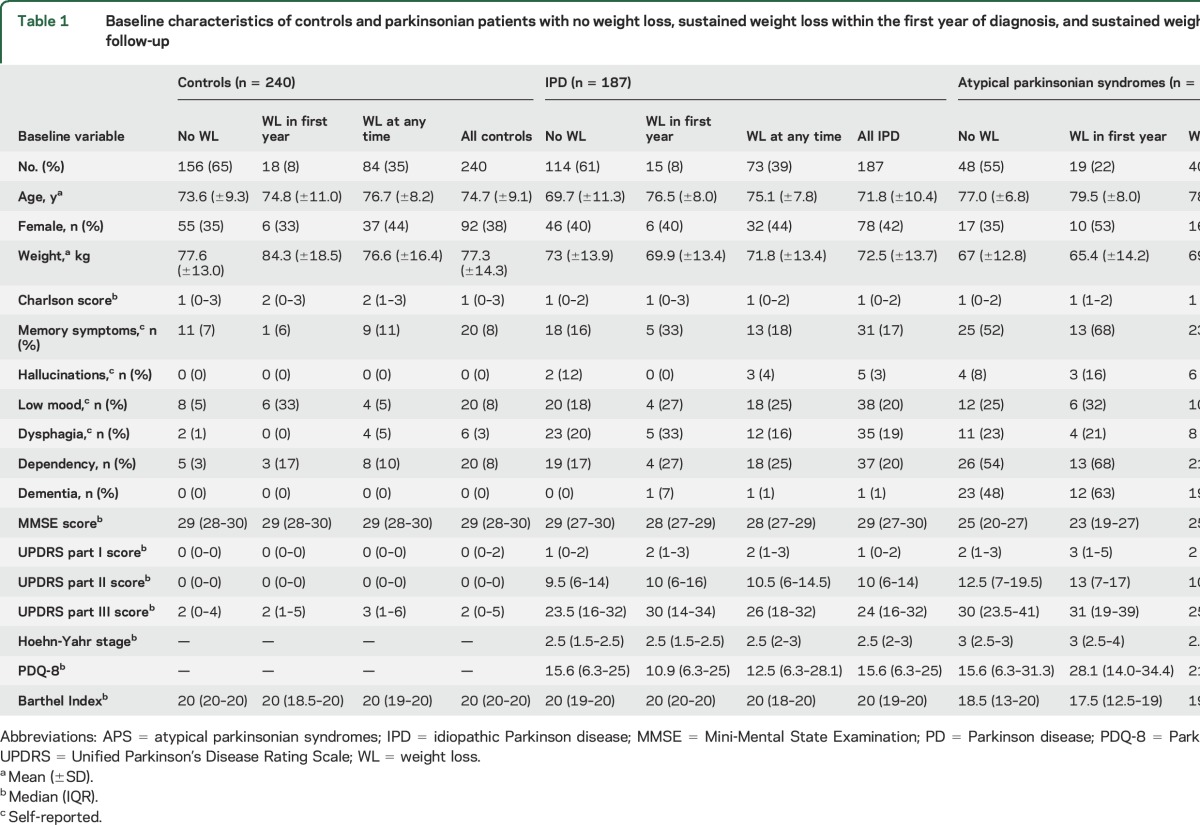
Baseline characteristics of controls and parkinsonian patients with no weight loss, sustained weight loss within the first year of diagnosis, and sustained weight loss at any time throughout follow-up

### Determinants of weight loss in parkinsonism.

The multivariable Cox regression model examining the determinants of weight loss was constructed for all 275 parkinsonian patients. Variables included and excluded from the models of outcomes of weight loss are listed in table 2. At diagnosis, parkinsonian patients (PD and atypical) who went on to develop sustained weight loss at any time after diagnosis were on average 4.5 years older and had a 1-point-higher median part I Unified Parkinson's Disease Rating Scale score than those without sustained weight loss (table 1).

**Table 2:**
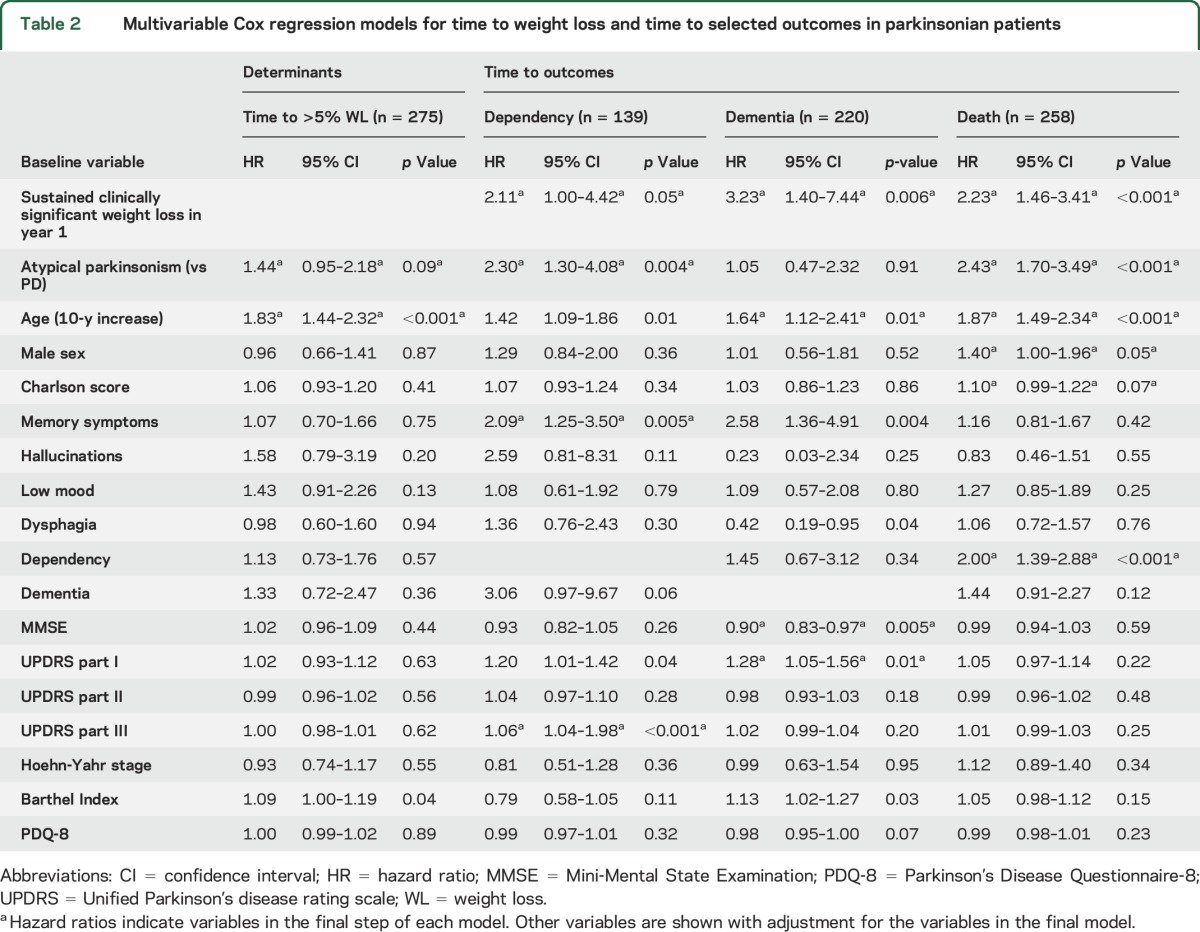
Multivariable Cox regression models for time to weight loss and time to selected outcomes in parkinsonian patients

After multivariable adjustment, only age was independently associated with developing sustained clinically significant weight loss at any time after parkinsonism diagnosis (hazard ratio [HR] 1.83, 95% confidence interval [CI] 1.44–2.32 for each 10-year increase in age) (table 2). There was an indication that atypical parkinsonism may be associated with greater weight loss than PD (HR 1.44, 95% CI 0.95–2.18). The association between lower dependency measured by the Barthel Index and weight loss after adjustment for age and diagnostic group (table 2) was probably spurious because of collinearity.

### Prognosis of clinically significant weight loss in parkinsonism.

In our parkinsonian cohort (n = 275), 85 were dependent at baseline, 43 developed dependency by year 1, 97 developed dependency during later follow-up, and 50 remained independent; 43 had dementia at baseline, 11 developed dementia by year 1, 57 developed dementia during follow-up, and 164 did not develop dementia. There were 162 deaths. The variables included and excluded from the models of outcomes of weight loss are listed in table 2.

Sustained clinically significant weight loss within the first year of diagnosis was independently strongly associated with subsequent dementia (HR 3.23, 95% CI 1.40–7.44) and mortality (HR 2.23, 95% CI 1.46–3.41) and was associated with dependency (HR 2.11, 95% CI 1.00–4.42, *p* = 0.05) (table 2). There was no evidence that the effect of sustained clinically significant weight loss on these outcomes was modified by parkinsonian diagnoses (*p* = 0.13, 0.93, and 0.20 for interaction in dependency, dementia, and mortality models, respectively). None of the associations changed if vascular parkinsonism was excluded.

## DISCUSSION

This study shows that people with parkinsonism had significantly lower body weight than controls at diagnosis. Weight loss was observed in all groups over time, but patients with PD lost weight more rapidly than controls, and those with atypical parkinsonism lost weight most rapidly. Age was the only independent baseline risk factor for sustained clinically significant weight loss in parkinsonism. Clinically significant weight loss within the first year of diagnosis was independently associated with subsequent dependency, dementia, and death after adjustment for potential confounders.

Lower body weight in patients with PD compared to nonparkinsonian controls has previously been reported.^3,19–21^ Two independent studies have observed weight loss occurring up to 10 years before parkinsonism diagnosis.^2,22^ These findings and ours suggest that weight loss is a feature of premotor and early motor parkinsonism, probably due to pathologic mechanisms predating motor symptom onset.

Like most previous studies,^1,2,20^ we found weight loss in PD to be greater than in controls. In a small 10-year longitudinal study, the proportion of clinically significant weight loss in patients with PD (n = 49) was double that among controls.^1^ Another study found that patients with PD had 4-fold higher odds of self-reported weight loss over 8 years.^20^ The observed weight loss over time in the PINE study is consistent with reports that weight loss in parkinsonism is associated with disease duration.^20^ However, weight measurements in later years of follow-up may be skewed by attrition due to mortality because those who survived longer were less likely to have early weight loss.

The finding that weight loss was greater in atypical parkinsonism than in PD is novel. This is in keeping with the fact that these are more aggressive diseases than PD and with previous studies in PD that show that weight loss is associated with greater severity of parkinsonism.^1,20^ This study complements data in other neurodegenerative disorders such as Alzheimer dementia and Huntington chorea, which are also associated with weight loss in early and later disease.^23,24^ This suggests that there may be mechanisms common to neurodegenerative disorders, possibly relating to increased catabolic cellular activity.

Similar to others, we found that age was independently associated with sustained clinically significant weight loss in parkinsonism.^1,3,5,10^ Unlike this study, others have found that weight loss was associated with female sex,^3,4,10,25^ disease severity,^1,10,20^ hallucinations,^1^ levodopa dose,^5,25^ dyskinesia,^19^ olfactory impairment,^26^ and comorbidities.^5^ The reason for this discrepancy may be in part that we studied only baseline predictors of weight loss and several of these features are only infrequently present at diagnosis. Further work to account for changing covariates over time (e.g., as time-dependent variables in Cox regression) may be useful. However, later measurement of predictive factors is less useful for early prediction of weight loss when targeted interventions could potentially be delivered.

We found that clinically significant weight loss within the first year of parkinsonism diagnosis was independently associated with dependency, dementia, and death. This effect was independent of diagnosis (PD or atypical), and there was no evidence of an interaction between weight loss and diagnosis. This suggests that the greater weight loss seen in atypical parkinsonism can be explained by the confounders that we adjusted for in the models. However, we still may not have detected a small difference because of a lack of power.

To the best of our knowledge, an association between weight loss and dependency has not previously been demonstrated. Our finding complements previous reports that weight loss and poor nutritional status in parkinsonism are associated with decreased quality of life,^5^ worsening disease severity,^6^ and dyskinesias,^26^ which are often accompanied by increased dependency.

Associations between weight loss and dementia in elderly populations have been reported but not in PD.^27,28^ Two small prospective studies of PD (n = 28 and 104) found that patients with weight loss and low body mass index had significantly worse cognitive function (after 1 and 3 years of follow-up, respectively).^3,7^

Two other studies have specifically investigated the association between weight loss and mortality in parkinsonism.^4,6^ One small retrospective audit (n = 55) found that weight loss was nonsignificantly associated with higher odds of mortality.^4^ Another large longitudinal study found no association between change in body mass index and survival, although this analysis had few deaths (76 of 1,673 patients).^6^

Although we have demonstrated an association between early weight loss and subsequent poor outcomes, our study was observational, so it cannot prove causation. However, even if weight loss is simply a marker of greater disease severity and progression or a manifestation of general frailty or other important comorbidities that worsen outcomes, it may nonetheless be useful clinically. To demonstrate a causal association, it would be necessary to perform an intervention trial aimed at preventing weight loss and to demonstrate that it resulted in improved outcomes. We are not aware of any such trials in parkinsonian disorders; indeed, there are currently no interventions proven to reduce weight loss in parkinsonism.^29^ Nevertheless, given the association of weight loss with poor outcomes and the low risk of harm, it is clearly important that the role of high-calorie (e.g., high fat and carbohydrate) interventions be investigated in the early stages of these diseases.

This study has several strengths. First, selection bias is likely to be low because we studied a population-based incident cohort in which efforts were made to identify all incident cases. There may have been more selection bias in the control group, but we have previously shown that the control group was not overly healthy.^30^ In addition, because of this design, the results are likely to be generalizable to the general population of PD, but there were relatively few young-onset patients because they make up a small proportion of the total population with PD. Second, a relatively large sample size (n = 275), with patients followed up prospectively from diagnosis with regular weight measurements and detailed characterization of clinical features, has provided more detailed data on predictors and outcomes of weight loss in parkinsonism and with longer follow-up than in any previous studies. The use of actual weight measurements from routinely calibrated scales improves on previous methods using self-reported weights.^20^ Third, we corrected for potential confounders and have studied important clinically relevant outcomes.

However, this study does have some limitations. First, some patients had missing weight data. These patients often had home visits because they were too frail to attend the clinic and thus may have been at greater risk of weight loss and poor outcome. This could have led to underestimation of differences between patients and controls and underestimation of the associations between weight loss and poor outcome. Second, data on other potential confounders, including intentional weight change, dietetic interventions, nutritional status, medication, and potential external stressors, were not available. Finally, sustained weight loss cannot be used prospectively as a predictor in early disease because, at the time of making the predictions, it is not known whether the weight loss will be sustained.

We have shown that parkinsonian patients have lower body weight and lose more weight over time than nonparkinsonian controls and that early clinically significant weight loss was independently associated with poor outcomes but that it is difficult to predict at baseline who will lose weight. While causality cannot be implied, close monitoring of weight seems advisable in parkinsonism because the identification of patients with early weight loss may have prognostic significance and potential for targeted intervention. Further work to investigate the effect of patient characteristics that develop over time in parkinsonism should be performed, including medication data, cumulative disease characteristics, and comorbidities. Targeted interventions to prevent or reverse weight loss in parkinsonism may improve outcomes, and there is therefore a strong case to investigate such interventions in randomized trials.

## GLOSSARY

CI: confidence interval
DSM-IV: *Diagnostic and Statistical Manual of Mental Disorders, 4th edition*
HR: hazard ratio
PD: Parkinson disease
PINE: Parkinsonism Incidence in North-East Scotland

## References

[1] Uc EY, Struck LK, Rodnitzky RL, Zimmerman B, Dobson J, Evans WJ. Predictors of weight loss in Parkinson's disease. Mov Disord 2006;21:930–936. 16534756 10.1002/mds.20837

[2] Chen H, Zhang SM, Hernan MA, Willett WC, Ascherio A. Weight loss in Parkinson's disease. Ann Neurol 2003;53:676–679. 12731005 10.1002/ana.10577

[3] Lorefält B, Ganowiak W, Pålhagen S, Toss G, Unosson M, Granérus AK. Factors of importance for weight loss in elderly patients with Parkinson's disease. Acta Neurol Scand 2004;110:180–187. 15285776 10.1111/j.1600-0404.2004.00307.x

[4] Walker R, Davidson M, Gray W. Gender differences in 1-year survival rates after weight loss in people with idiopathic Parkinson's disease. Int J Palliat Nurs 2012;18:35–39. 22306718 10.12968/ijpn.2012.18.1.35

[5] Akbar U, He Y, Wu S, et al. Weight loss and impact on quality of life in Parkinson’s disease. PLoS One 2015;10:e0124541. 25938478 10.1371/journal.pone.0124541 PMC4418600

[6] Wills AM, Perez A, Wang J, et al. Association between change in body mass index, Unified Parkinson's Disease Rating Scale scores, and survival among persons with Parkinson disease: secondary analysis of longitudinal data from NINDS Exploratory Trials in Parkinson Disease Long-Term Study 1. JAMA Neurol 2016;73:321–328. 26751506 10.1001/jamaneurol.2015.4265 PMC5469290

[7] Kim HJ, Oh ES, Lee JH, et al. Relationship between changes of body mass index (BMI) and cognitive decline in Parkinson's disease (PD). Arch Gerontol Geriatr 2012;55:70–72. 21763014 10.1016/j.archger.2011.06.022

[8] Sheard JM, Ash S, Mellick GD, Silburn PA, Kerr GK. Improved nutritional status is related to improved quality of life in Parkinson's disease. BMC Neurol 2014;14:212–220. 25403709 10.1186/s12883-014-0212-1 PMC4237731

[9] Sharma JC, Vassallo M. Prognostic significance of weight changes in Parkinson's disease: the Park-weight phenotype. Neurodegener Dis Manag 2014;4:309–316. 25313987 10.2217/nmt.14.25

[10] Wills A-M, Ruosha L, Perez A, Ren X, Boyd J. Predictors of weight loss in early treated Parkinson's disease from the NET-PD LS-1 cohort. J Neurol 2017;264:1746–1753. 28712000 10.1007/s00415-017-8562-4 PMC5789795

[11] Taylor KS, Counsell CE, Harris CE, Gordon JC, Smith WC. Pilot study of the incidence and prognosis of degenerative Parkinsonian disorders in Aberdeen, United Kingdom: methods and preliminary results. Mov Disord 2006;21:976–982. 16570298 10.1002/mds.20866

[12] Caslake R, Taylor K, Scott N, et al. Age-, gender-, and socioeconomic status-specific incidence of Parkinson's disease and parkinsonism in North East Scotland: the PINE study. Parkinsonism Relat Disord 2013;19:515–521. 23462482 10.1016/j.parkreldis.2013.01.014

[13] Hughes AJ, Daniel SE, Kilford L, Lees AJ. Accuracy of clinical diagnosis of idiopathic Parkinson's disease: a clinico-pathological study of 100 cases. J Neurol Neurosurg Psychiatry 1992;55:181–184. 1564476 10.1136/jnnp.55.3.181 PMC1014720

[14] Bouras EP, Lange SM, Scolapio JS. Rational approach to patient with unintentional weight loss. Mayo Clin Proc 2001;76:923–929. 11560304 10.4065/76.9.923

[15] Schwab RS, England AC. Projection technique for evaluating surgery in Parkinson's disease. In: Gillingham FJ, Donaldson MC, eds. Third Symposium on Parkinson's Disease. Edinburgh: E & S Livingston; 1969:152–157.

[16] Caslake R, Summers F, McConachie D, et al. The Mini-Mental Parkinson's (MMP) as a cognitive screening tool in people with Parkinson's disease. Curr Aging Sci 2013;6:273–279. 23773030 10.2174/18746098112059990030

[17] Macleod AD, Goddard H, Counsell CE. Co-morbidity burden in Parkinson's disease: comparison with controls and its influence on prognosis. Parkinsonism Relat Disord 2016;28:124–129. 27210815 10.1016/j.parkreldis.2016.05.013 PMC4925465

[18] Peduzzi P, Concato J, Feinstein AR, Holford TR. Importance of events per independent variable in proportional hazards regression analysis, II: accuracy and precision of regression estimates. J Clin Epidemiol 1995;48:1503–1510. 8543964 10.1016/0895-4356(95)00048-8

[19] Markus HS, Cox M, Tomkins AM, Stern GM. Increased prevalence of undernutrition in Parkinson's disease and its relationship to clinical disease parameters. J Neural Transm Park Dis Dement Sect 1993;5:117–125. 8333907 10.1007/BF02251202

[20] Beyer PL, Palarino MY, Michalek D, Busenbark K, Koller WC. Weight change and body composition in patients with Parkinson's disease. J Am Diet Assoc 1995;95:979–983. 7657912 10.1016/S0002-8223(95)00269-3

[21] Van der Merck MA, Dicke HC, Uc EY, et al. Body mass index in Parkinson's disease: a meta-analysis. Parkinsonism Relat Disord 2012;18:263–267. 22100523 10.1016/j.parkreldis.2011.10.016

[22] Logroscino G, Sesso HD, Paggenbarger RS, Lee IM. Body mass index and risk of Parkinson's disease: a prospective cohort study. Am J Epidemiol 2007;166:1186–1190. 17709328 10.1093/aje/kwm211

[23] Besser LM, Gill DP, Monsell SE, et al. Body mass index, weight change, and clinical progression in mild cognitive impairment and Alzheimer disease. Alzheimer Dis Assoc Disord 2014;28:36–43. 24126214 10.1097/WAD.0000000000000005 PMC3945175

[24] Djousse L, Knowlton B, Cupples LA, Marder K, Shoulson I, Myers RH. Weight loss in early stage of Huntington's disease. Neurology 2002;59:1325–1330. 12427878 10.1212/01.wnl.0000031791.10922.cf

[25] Sharma JC, Turton J. Olfaction, dyskinesia and profile of weight change in Parkinson's disease: identifying neurodegenerative phenotypes. Parkinsonism Relat Disord 2012;18:964–970. 22682755 10.1016/j.parkreldis.2012.05.004

[26] Sharma JC, Macnamara L, Hasoon M, Vassallo M, Ross I. Cascade of levodopa dose and weight related dyskinesia in Parkinson's disease (LD–WD–PD cascade). Parkinsonism Relat Disord 2006;12:499–505. 16935018 10.1016/j.parkreldis.2006.07.002

[27] Stewart R, Masaki K, Xue QL, et al. A 32-year prospective study of change in body weight and incident dementia: the Honolulu-Asia Aging Study. Arch Neurol 2005;62:55–60. 15642850 10.1001/archneur.62.1.55

[28] Atti AR, Palmer K, Volpato S, Winblad B, De Ronchi D, Fratiglioni L. Late-life body mass index and dementia incidence: nine-year follow-up data from the Kungsholmen Project. J Am Geriatr Soc 2008;56:111–116. 18028342 10.1111/j.1532-5415.2007.01458.x

[29] Payne C, Wiffen PJ, Martin S. Interventions for fatigue and weight loss in adults with advanced progressive illness. Cochrane Database Syst Rev 2012;1:CD008427. 22258985 10.1002/14651858.CD008427.pub2

[30] Taylor KSM, Gordon JC, Harris CE, Counsell CE. Recruitment bias resulted in poorer overall health status in a community-based control group. J Clin Epidemiol 2008;61:890–895. 18468853 10.1016/j.jclinepi.2007.10.020

